# Family relationships and personality traits of postural tachycardia syndrome

**DOI:** 10.3389/fped.2025.1543200

**Published:** 2025-04-25

**Authors:** Meiko Matsui, Seiji Yoshida, Hidetaka Tanaka, Ginroku Yamawake, Yusuke Kurooka, Yoshitaka Ota, Atsuko Kubo, Midori Mizutani, Akira Ashida

**Affiliations:** ^1^ Department of Pediatrics, Osaka Medical and Pharmaceutical University Hospital, Takatsuiki, Japan; ^2^ OD Hypotension Clinic Tanaka, Osaka, Japan; ^3^ Department of Pediatrics, Saiseikai Suita Hospital, Suita, Japan; ^4^ Department of Pediatrics, Saiseikai Ibaraki Hospital, Ibaraki, Japan; ^5^ Department of Pediatrics, Hokusetsu General Hospital, Takatsuki, Japan

**Keywords:** orthostatic dysregulation, postural tachycardia syndrome, parent–child relationships, personality traits, autonomic neuron function

## Abstract

**Introduction:**

Orthostatic intolerance is the name of the disease in Europe and the United States; however, in Japan, it is commonly known as orthostatic dysregulation (OD). OD is a physical disorder caused by failure of the body's compensatory regulatory mechanism to adapt to changes in circulatory dynamics during orthostasis; however, in many cases, the influence of psychosocial factors has been noted. The mother–child relationship is a major psychosocial factor in children, and it has been reported that parent–child patterns associated with OD are often excessive parental interference and child over-adaptation. This study focused on the psychological and physical factors of OD and assessed the parent–child relationship patterns among children with postural tachycardia syndrome (POTS), a subtype of OD, and examined how they relate to the child's personality traits and autonomic neuron function.

**Methods:**

A total of 36 six children diagnosed with POTS (mean age 13.5 ± 0.9 years) were compared with the results of the following questionnaires on parent–child relationships, personality traits, and the autonomic neuron function test: Family diagnostic test (a diagnostic test of parent–child relationship) for mothers and children, AN-EGOGRAM (the egograms that can be adapted to childhood and adolescence, when the ego is in the process of developing and growing), and a frequency analysis of heartrate and blood pressure variability for the children.

**Results:**

Assessments of the mother–child relationship patterns among children with POTS found significant associations between maternal “strict discipline” and children's negative feelings, excessive parental intervention and children's susceptibility to stress, and the degree of children's “feeling of rejection” and lower supine vasomotor sympathetic nerve activity. The findings also suggested that children with POTS typically exhibited lower scores in critical parent and free child personality traits, but higher scores in adapted child (AC). Notably, mothers of children with high AC scores reported less dissatisfaction with their spouses regarding childcare.

**Discussion:**

In the parent–child relationship in POTS, an association was observed between maternal “strict discipline” and children's negative feelings, suggesting that a less strict disciplinary approach may contribute to a more positive parent–child relationship.

## 1 Introduction

The condition commonly known as orthostatic intolerance in the Europe and the United States is referred to as orthostatic dysregulation (OD) in Japan, particularly in pediatrics. It occurs due to the failure of the body's compensatory mechanisms to adapt to circulatory changes during orthostasis, resulting in a range of circulatory symptoms such as dizziness, palpitations, reduced systolic blood pressure, decreased cerebral blood flow, and increased heart rate, as well as autonomic symptoms, including poor appetite, nausea, and abdominal pain (1). Children with OD often experience these symptoms for an extended period, with psychosocial stress frequently exacerbating their condition and interfering with daily life. Therefore, regardless of whether the onset is triggered by physical or psychosocial factors, effective treatment of OD requires a combination of physical treatment and psychological support, as these factors are closely interconnected (2).

Adolescents are prone to developing OD, with a prevalence of approximately 5% among primary school students and 10% among junior and senior high school students, including mild cases, with an estimated 120,000 children in each grade (approximately 700,000 junior and senior high school students in total) (3). OD is more likely to co-occur with school refusal owing to poor health conditions in the morning, which make it difficult to attend school. Approximately half of the children who require medical treatment experience school refusal (1). Children who are absent from school with physical complaints are often regarded by others as “lazy” or “problematic,” rather than psychosomatic. Consequently, Japanese children with OD have been reported to have psychosocial problems in terms of personality as well as interpersonal and family relationships (1).

The Japanese Society of Psychosomatic Pediatrics, based on a large number of studies accumulated over nearly half a century, categorized OD into four different circulatory subsets: (i) instantaneous orthostatic hypotension, (ii) postural tachycardia syndrome (POTS), (iii) vasovagal syncope, and (iv) delayed orthostatic hypotension (1). The causes of POTS include loss of plasma volume (includes absolute and relative hypovolemia) (4) and insufficient venous constriction or hyperadrenergic response to orthostatic stress. The POTS criteria are defined as follows: increase in heartrate during standing (≧35 beats/min) or a heart rate during active standing of ≧115 beats/min. It involves marked tachycardia in an upright posture without obvious hypotension, and symptoms such as light-headedness, fatigue, and headaches (1).

Specific personality traits have also been reported among adolescents with POTS; they are characterized as being “high achievers,” typically have excellent grades, and are successfully involved in multiple extracurricular activities (it could be postulated that the hyper-stimulated nervous system of a high achiever may be more prone to developing neurotransmitter abnormalities leading to POTS) (5). In addition, psychosocial factors have been suggested to play a role in the onset and exacerbation of symptoms in OD (1). Among these, the parent–child relationship, particularly the mother–child dynamic, has been identified as a major factor. In addition, children with OD often exhibit over-adaptive behavior and are subject to excessive parental involvement (1).

However, family relationships and personality tendencies in children with POTS remain unclear. Clarifying the parent–child relationship, the child's personality traits, and autonomic neuron function can aid in the development of effective interventions to address psychosocial factors. Therefore, this study assessed the patterns of parent–child relationships among children with OD and examined how they relate to the child's personality traits and autonomic neuron function.

## 2 Methods

We investigated 36 children (18 boys and 18 girls; age range 12–16 years; mean age 13.5 ± 0.9 years) who visited the pediatrics department of the Osaka Medical and Pharmaceutical University Hospital between March 2022 and March 2024 and were diagnosed with POTS using the standing test. The children and their parents gave their consent to participate in the study. Children with comorbidities identified through physical examinations or blood tests, as well as those without mothers, were excluded from the study. Autonomic neuron function was assessed using a frequency analysis of blood pressure and heart rate variability data from the standing test at the first visit. The standing test was conducted in a constant test environment, considering the effect on autonomic neuron function. The patients were quietly seated for 15 min in a waiting room before the actual measurement started. The test was performed in the morning in a soundproof room, with the temperature maintained at 23°C–25°C. The diagnosis of POTS was defined as 10 min in the resting supine position followed by 10 min in the upright position, and a heart rate increase of more than 35 bpm or tachycardia of more than 115 bpm after 3 min in the upright position (1). Psychological tests included the family diagnostic test (FDT) for mothers and AN-EGOGRAM for children. Details of the autonomic neuron function assessment and psychological testing are described below.

### 2.1 Evaluation of autonomic neuron function

We assessed autonomic neuron function based on the results of a frequency analysis of heart rate and blood pressure variability during the standing test. POTS is a complex, multi-system chronic disease of the autonomic nervous system (4), and autonomic neuron function assessment is useful for clinical evaluation. Frequency analysis evaluated the high-frequency (HF; 0.15–0.4 Hz) and low-frequency (LF; 0.04–0.15 Hz) components of heart rate (RR interval) and blood pressure variability. It is generally considered that the HF component of the RR interval variability (RR-HF) is mediated by the cardiac parasympathetic tone. The LF component of the RR interval variation divided by the HF component (RR-LF/HF) is considered an indicator of cardiac sympathetic tone, whereas the LF component of blood pressure variability is considered an indicator of vasomotor sympathetic activity (6, 7). The LF component of diastolic blood pressure variability (DBP-LF) was assessed as a function of the vasomotor sympathetic function. Spectral analysis using the maximum entropy method (8) (MemCalc for Windows, version 1.2; Suwa Trust, Tokyo, Japan) was applied to the time-series data for each variable.

### 2.2 FDT

The FDT was used to examine family relationships, particularly parent–child relationships. The FDT questionnaire has been standardized and found to be reliable with Cronbach's alpha coefficients in the range of 0.54–0.89. Some alpha coefficients are low, but this is due to the number of items in each scale (five items). An alpha coefficient at this level is considered sufficiently reliable (9). The FDT can be considered to measure an emotional aspect of how both the parent and the child perceive the parent–child relationship. There are two types of questionnaires for children and parents, each consisting of 60 items on eight scales for children and 40 items on seven scales for parents; the terms and their contents are shown in Tables 1 and 2, respectively. A 5-point scale ranging from “strongly disagree” to “strong agree” is used to answer each question. The total score for each scale is calculated, and percentile values are obtained using the percentile conversion table in the manual.

**Table 1:** Scale structure and content of the children's FDT.

Item	Content
Feeling of rejection	Degree to which children feel they are rejected by their parents.
Positive avoidance	Degree to which children avoid contact or being involved with their parents as much as possible.
Psychological interference	Degree to which children feel their privacy is being violated by their parents.
Strict discipline	Degree to which parental discipline is perceived as strict.
Discrepancy between parents	Degree to which children perceive differences in parental attitudes toward childcare and education, and mutual dissatisfaction.
Achievement request	Degree to which children feel they are under pressure from their parents.
Feeling of acceptance	Degree to which children feel their parents trust and accept them.
Emotional closeness	Degree to which children are emotionally accepting of their parents.

**Table 2:** Scale structure and content of the parents’ FDT.

Item	Content
Uninterested	Degree to which parents show a lack of interest in their children.
Childcare anxiety	Degree to which parents feel anxious and unconfident.
Discrepancy in marriage	Degree of dissatisfaction with the partner, especially regarding childcare.
Strict discipline	Degree to which strict discipline is exercised at home.
Achievement request	Degree of over-expectation of children by the parents.
Non-involvement	Degree to which parents do not interfere in children's behavior.
Basic acceptance	Degree to which parents are accepting of their children.

### 2.3 AN-EGOGRAM (ego state assessment)

The egogram was developed by Dusay based on the transactional analysis theory founded by Erick Burne to capture ego states functionally and visualize them intuitively (10). The egogram for adults has been standardized as a Tokyo University Egogram (TEG); however, a new, modified version for children is the AN-EGOGRAM (11).

The egogram captures ego states on five scales. These five scales can be simply described as critical parent (CP; “strictness”), nurturing parent (NP; “gentleness”), adult (A; “calmness”), free child (FC; “free-spiritedness”), and adapted child (AC; “obedience”). A more detailed summary of the functions of each ego is provided in Table 3.

**Table 3:** Characteristics of the five egograms.

Scale	High	Low
CP (ego trying to fulfill its social function)	Pursue the ideal High demands on others	Laid back Not clear about what they want to say
NP (ego that feeds and nurtures others)	Kindness and consideration Overprotectiveness and excessive interference	Plain, unemotional Cold
A (rational ego that thinks logically)	Make decisions objectively based on the facts Mechanical thinking and lacking human touch	Simple, good-natured, and carefree Unplanned
FC (ego that is free to be its natural self)	Innocent, inquisitive, creative Self-centered, selfish	Meek, straightforward, and cautious Sometimes seems unmotivated and gloomy
AC (ego that is sensitive to others’ facial expressions and adapts)	Highly cooperative with honor-oriented temperament Tends to be reserved, hides emotions and endure	Reveal the real self, vivacious, proactive Self-centered, selfish

These five egos are present in everyone, and their behavior changes according to the balance of strength and weaknesses (11). The scores for each of the five egos on the egogram are summed, and the T-score conversion table is used to determine the T-score from its raw score. A score in the range of 43–57 is considered average, >58 is high, and <42 is low (11).

Statistical analyses of all data were performed using JMP Pro 17. Spearman's rank order correlation coefficient was used to assess the correlation between the two groups, and Wilcoxon's rank-sum test was used to assess differences in median values. The significance level for each test was set at *p* < 0.05.

## 3 Results

For each FDT item for mothers and children, significant correlations were found between the mothers’ strict discipline and each of the children's items, as shown in Table 4.

**Table 4:** Correlation between mother and child FDT items.

Mother	Child	Spearman's rank correlation coefficient (*ρ*)	*p*
Strict discipline	Feeling of rejection	0.52	<0.01
	Positive avoidance	0.58	<0.001
	Psychological interference	0.34	<0.05
	Strict discipline	0.52	<0.01
	Discrepancy between parents	0.42	<0.05
	Achievement request	0.31	0.06
	Feeling of acceptance	−0.63	<0.001
	Emotional closeness	−0.52	<0.01

Regarding the correlations between individual mother–child FDT items and the child's autonomic neuron function, greater maternal non-involvement in the child's behavior was associated with higher suRR-HF, while a stronger sense of maternal rejection experienced by the child was linked to lower suDBP-LF (Table 5).

**Table 5:** Correlation between each FDT item and autonomic neuronal function.

FDT	ANS	Spearman's rank correlation coefficient (*ρ*)	*p*
Non-involvement	suRR-HF	0.40	<0.05
Feeling of rejection	suDBP-LF	−0.33	<0.05

ANS, autonomic neuron function; suRR-HF, supine high-frequency component of the RR interval variability; suDBP-LF, supine low-frequency component of the DBP variability.

**Figure 1:**
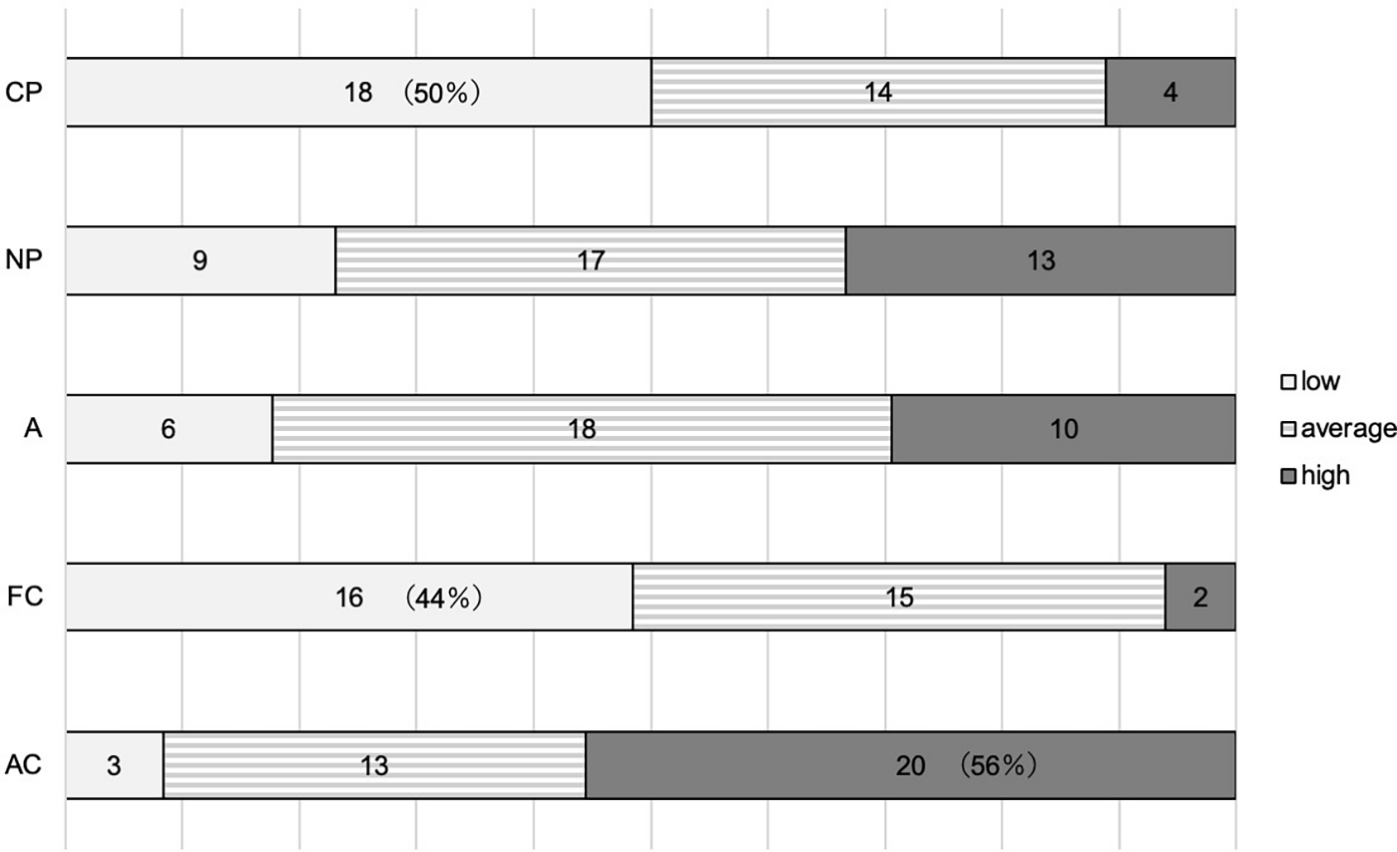
The low-egogram group is indicated by white bars, the average egogram group by striped bars, and the high-egogram group by gray bars. CP, critical parent; NP, nurturing parent; A, adult; FC, free child; AC, adapted child.

The percentages of each egogram are shown in Figure 1. Approximately 50% of the children scored low on CP and 44% scored low on FC. Nearly 56% of the children also had a higher AC.

The low and other CP and FC groups, and the high and other AC groups in the egogram were compared. The results showed that the higher AC group had lower marital discrepancy (*p* = 0.045).

## 4 Discussion

This study examined parent–child relationship patterns, the personality traits of children with POTS, and their autonomic neuron function, to form the basis for the development of interventions targeting psychosocial factors in affected children.

There was a positive correlation between maternal “strict discipline” and children's “feeling of rejection,” “psychological interference,” and “positive avoidance” among the FDT items for mothers and children. In contrast, there was a negative correlation between maternal “strict discipline” and children's “feeling of acceptance” and “emotional closeness.” Although moderation in strict discipline is generally considered preferable (10), in the context of POTS, maternal “strict discipline” was associated with negative feelings in children, suggesting that a less rigid approach may foster a better parent–child relationship.

There was a positive correlation between parental “non-involvement” and cardiac parasympathetic function in the child's supine position, as for FDT items and autonomic neuron function. As the cardiac parasympathetic function decreases under stressful conditions (12), children with POTS are more likely to experience parental interference as stressful, and parents need to be careful in their involvement with their children. In addition, there was a negative correlation between children's “feeling of rejection” and vasomotor sympathetic activity in the supine position. Vasomotor sympathetic dysfunction causes hypotension, and in this study, there was a positive correlation between scores of systolic blood pressure in the supine position and children's “feelings of rejection” (*ρ* = −0.33, *p* = 0.05), indicating that stronger feelings of rejection were related to more hypotension. Hypotension has been reported to affect fatigue (13) and may be related to fatigue in the supine position in children with POTS.

Egogram analysis suggested that children with POTS tended to have lower CP and FC scores, and higher AC scores. Children with lower CP scores are often gentle and avoid asserting their opinions or values. Lower FC scores suggest they are honest and cautious, but prone to suppressing emotional responses and avoiding conflict in interpersonal relationships. Those with higher AC scores tend to be cooperative but also tend to suppress their true selves. These personality traits are commonly observed in children with POTS. It has been pointed out that children with OD are more likely to be over-adapted (1) as they worry about what others think of them and are prone to feelings of inferiority.

In psychosomatic disorders, egograms are thought to reflect psychological states (14). Studies of adult patients with psychosomatic disorders have shown that individuals with irritable bowel syndrome had higher CP and AC scores, while those with anorexia nervosa had higher CP and AC scores but lower FC scores (14). This suggests a similarity in egogram patterns between children with POTS and adults with psychosomatic disorders, namely high AC and low FC scores, although there are differences in CP scores. The lower CP in children with POTS may reflect their tendency to be generous, unassertive, and adaptable to their surroundings.

Comparisons between egogram profiles and FDT scores revealed a negative correlation between the child's AC score and parental “discrepancy in marriage.” Children with higher AC scores tend to be perceptive of others’ emotions and behave flexibly. In addition, when marital discrepancy scores are low, parents are more likely to trust their spouses, which enhances their confidence as parents and encourages them to treat their children with warmth and attentiveness (10). Lower dissatisfaction with a spouse regarding childcare is associated with a more confident and receptive parenting style that respects the child’s needs and assertions. As a result, we speculate that children develop more empathy, adopt a more generous ego state toward others, and may be more willing to compromise.

## 5 Limitations

As autonomic neuron function varies between races (15), there are differences in diagnostic criteria. The diagnostic criterion for pediatric POTS in Europe and the United States is an increase in heart rate of 40 bpm or more, while in Japan it is 35 bpm or more. The parent–child relationship assessment in this study was conducted using the FDT with mothers and children, as only mothers and children visited the hospital. In the future, we intend to examine this relationship with fathers as well. It is advisable to interpret these findings with caution as the egogram patterns are not absolute or invariant and because AN-EGOGRAM results are only a reflection of the ego state and parent–child relationship at the time of first visit.

## 6 Conclusions

This study assessed mother–child relationship patterns in children with POTS and found significant associations between maternal “strict discipline” and the child’s negative feelings, excessive parental intervention and the child’s susceptibility to stress, and the degree of children's “feeling of rejection” and lower supine vasomotor sympathetic nerve activity. The findings also suggested that children with POTS tend to exhibit personality traits characterized by lower CP and FC scores, and higher AC scores. In addition, mothers of children with high AC scores reported less dissatisfaction with their spouses regarding childcare.

## Data Availability

The raw data supporting the conclusions of this article will be made available by the authors, without undue reservation.
